# Serological Responses and Predictive Factors of Booster COVID-19 Vaccines in Patients with Hematologic Malignancies

**DOI:** 10.3390/jcm12175647

**Published:** 2023-08-30

**Authors:** Chien-Tzu Huang, Ching-Ping Lee, Tzu-Yin Chen, Yi-Chang Liu, Shih-Feng Cho, Jeng-Shiun Du, Ming-Lung Yu, Chung-Feng Huang, Sheng-Fan Wang, Hui-Hua Hsiao

**Affiliations:** 1 Division of Hematology and Oncology, Department of Internal Medicine, Kaohsiung Medical University Hospital, Kaohsiung Medical University, Kaohsiung 807, Taiwan; gankay18@hotmail.com (C.-T.H.); 890218@kmuh.org.tw (C.-P.L.); ycliu@cc.kmu.edu.tw (Y.-C.L.); sifong96@gmail.com (S.-F.C.); ashiun@gmail.com (J.-S.D.); 2 Graduate Institute of Clinical Medicine, College of Medicine, Kaohsiung Medical University, Kaohsiung 807, Taiwan; 3 Department of Nursing, Kaohsiung Medical University Hospital, Kaohsiung Medical University, Kaohsiung 807, Taiwan; zuin1228@gmail.com; 4 Division of Hepatobiliary, Department of Internal Medicine and Hepatitis Center, Kaohsiung Medical University Hospital, Kaohsiung Medical University, Kaohsiung 807, Taiwan; fish6069@gmail.com (M.-L.Y.); fengcheerup@gmail.com (C.-F.H.); 5 School of Medicine and Doctoral Program of Clinical and Experimental Medicine, College of Medicine and Center of Excellence for Metabolic Associated Fatty Liver Disease, National Sun Yat-sen University, Kaohsiung 804, Taiwan; 6 Ph.D. Program in Translational Medicine, College of Medicine, Kaohsiung Medical University and Academia Sinica, Kaohsiung 807, Taiwan; 7 Center for Tropical Medicine and Infectious Disease Research, Kaohsiung Medical University, Kaohsiung 807, Taiwan; kmuwasf1234@gmail.com; 8 Department of Medical Laboratory Science and Biotechnology, Kaohsiung Medical University, Kaohsiung 807, Taiwan

**Keywords:** coronavirus disease 2019, booster vaccines, hematologic malignancies, B-cell-targeted agents, hypogammaglobinemia

## Abstract

Patients with hematologic malignancies are reported to have a more severe course of coronavirus disease 2019 (COVID-19) and be less responsive to vaccination. In this prospective study, we aimed to evaluate the serological responses to booster COVID-19 vaccines of Taiwanese patients with hematologic malignancies and identify potential predictive markers for effective neutralizing immunity. This study enrolled 68 patients with hematologic malignancies and 68 age- and gender-matched healthy control subjects who received three doses of vaccination against severe acute respiratory syndrome coronavirus 2 (SARS-CoV-2) from 1 January 2022 to 31 October 2022. The SARS-CoV-2 immunoglobulin G (IgG) spike antibody level was measured with the Abbott assay. The effective neutralization capacity was defined as an anti-spike IgG level of ≥4160 AU/mL. Among the 68 patients with hematologic malignancies, 89.7% achieved seroconversion after booster doses. Seven patients with actively treated lymphoma remained seronegative and had the lowest humoral responses among patients with different types of hematologic malignancies. Despite comparable antibody titers between patients and healthy individuals, rates of effective neutralization (66.2% vs. 86.8%, respectively; *p* = 0.005) were significantly reduced in patients with hematologic malignancies. In a multivariate analysis, the independent predictors for effective neutralization were a lack of B-cell-targeted agents within six months of vaccination (odds ratio, 15.2; 95% confidence interval, 2.7–84.2; *p* = 0.002) and higher immunoglobulin levels (odds ratio, 4.4; 95% confidence interval, 1.3–14.7; *p* = 0.017). In conclusion, the majority of patients with hematologic malignancies achieved seroconversion after booster vaccination. Patients with ongoing B-cell depletion and hypogammaglobinemia were identified as having negative predictive markers for effective neutralization.

## 1. Introduction

Coronavirus disease 2019 (COVID-19), caused by severe acute respiratory syndrome coronavirus 2 (SARS-CoV-2), emerged as an ongoing global pandemic in 2020. As of 20 March 2023, a total of 10,236,886 confirmed cases with 18,803 deaths have been reported by the Taiwan Centers for Disease Control. In Taiwan, more than 90% of COVID-19-related deaths have occurred among patients with comorbidities [1]. In addition to increasing age, comorbidities including obesity, diabetes, cardiac disease, respiratory disease, kidney disease, dementia, and malignancies are associated with a higher risk of mortality [2,3].

COVID-19 poses a great threat to patients with hematologic malignancies, accounting for severe illness and a high mortality rate of approximately 30% in the prevaccine era [4,5]. A large study conducted by the National Health Service in England showed that patients diagnosed with hematologic malignancies in the previous five years had a 2.5- to 3-fold increased risk of COVID-19-related death [3]. After the availability of antivirals, monoclonal antibodies, and vaccines against SARS-CoV-2, the mortality rate of these patients declined significantly; however, it was still notably higher than that of the overall population [6]. The reasons for the poor outcome are likely multifactorial, including immune dysfunction because of both underlying malignancies and anticancer therapy, cancer progression resulting from treatment delays, and suboptimal vaccine responses.

Previous studies have reported the limited responses of COVID-19 vaccines in patients with hematologic malignancies, especially in those with lymphoma or chronic lymphocytic leukemia (CLL) [7,8]. In a recent systematic review and meta-analysis involving over 20,000 hematologic malignancy patients [9], immunogenicity following COVID-19 vaccination was found to be substantially impaired, with a seroconversion rate of only 67.7% after two doses of vaccines, as opposed to healthy controls, of whom 98.7% were seropositive following primary vaccination. Treatment modalities such as chimeric antigen receptor T-cells, anti-CD20 monoclonal antibodies, Bruton tyrosine kinase (BTK) inhibitors, phosphatidylinositol-3 kinase (PI3K) inhibitors, B-cell lymphoma 2 (BCL2) inhibitors, and Janus kinase 2 (JAK2) inhibitors were associated with a lower percentage of seroconversion. The proportion of neutralizing antibody development and cellular immune responses against SARS-CoV-2 was also significantly lower in patients with hematologic malignancies compared to healthy controls. Additionally, the pooled booster-induced seroconversion rates ranged from 23% to 59% among seronegative patients following primary vaccination [10,11,12,13,14,15,16,17,18,19]. The responses varied depending on the type of cancer and therapy. The humoral immunity of booster vaccines in these patients from Asia is still uncertain.

In this prospective study, we aimed to evaluate serological responses and potential predictive markers for effective neutralization of booster COVID-19 vaccines in Taiwanese patients with hematologic malignancies.

## 2. Materials and Methods

### 2.1. Participants

From 7 January 2022, Taiwanese patients with a history of hematologic malignancies were prioritized to receive a booster dose of the COVID-19 vaccine at least 12 weeks after the primary two-dose vaccination. There are four approved vaccines against SARS-CoV-2 in Taiwan: a nonreplicating viral vector vaccine (AstraZeneca (AZ)), two messenger RNA (mRNA) vaccines (Moderna, Cambridge, MA, USA; BioNTech, Mainz, Germany), and a protein subunit vaccine (Medigen COVID-19 vaccine (MVC)).

Participants who received three doses of COVID-19 vaccination were prospectively recruited in a medical center in Taiwan from 1 January 2022 to 31 October 2022. Eligibility criteria for the study were as follows: (1) adult patients ≥ 20 years old; (2) a history of hematologic malignancies; (3) received three doses of a nationally approved COVID-19 vaccine; and (4) no known history of SARS-CoV-2 infection. Information about previous SARS-CoV-2 infection and contact histories was collected during the case interview via a well-designed questionnaire. Participants with a prior infection history or a positive contact history for COVID-19 were excluded from the study. Our study also enrolled healthy volunteers without known history of malignancies, who received three doses of COVID-19 vaccination and served as a control group in order to facilitate a comparison of postbooster serological responses with the patients. We used an age- and gender-matched analysis to lessen the influence of baseline demographic data on antibody titers.

The study was conducted in accordance with the Declaration of Helsinki and approved by the Institutional Review Board of Kaohsiung Medical University Hospital (KMUHIRB-E(I)-20210273). All patients provided informed consent.

### 2.2. Laboratory Analyses and Clinical Parameters

Blood samples and antibody titers were checked 1–3 months after the administration of booster COVID-19 vaccines. The SARS-CoV-2-immunoglobulin G (IgG) spike antibody level was measured using the Abbott assay (SARS-CoV-2 IgG II), which is highly correlated with the WHO International Standard (binding antibody unit (BAU)) (Abbott: BAU/mL = 0.142 × AU/mL) [20]. Per that definition, an IgG level ≥ 50 AU/mL was considered as a positive antibody response, meeting the criteria for seroconversion. Potentially effective neutralization capacity was defined as having an anti-spike IgG level ≥ 4160 AU/mL [20,21]. Anti-SARS-CoV-2 IgG level, rates of seroconversion, and effective neutralization were further analyzed as outcomes of our study.

In addition, serum levels of total IgG, IgA, and IgM were collected and measured with immunoturbidimetric assays. IgG, IgA, and IgM levels were divided into two subgroups, classified as higher immunoglobulin level (IgG ≥ 550 mg/dL, IgA ≥ 80 mg/dL, and IgM ≥ 40 mg/dL) and lower immunoglobulin level (IgG < 550 mg/dL, IgA < 80 mg/dL, or IgM < 40 mg/dL) in accordance with previous articles [22]. The clinical parameters were obtained from the predesigned questionnaire and the medical records, including demographic characteristics, comorbidities, cancer status, anticancer therapy, and vaccination types. Active treatment was defined as a time interval between the latest anticancer therapy and vaccination of less than six months.

### 2.3. Statistical Analysis

Baseline demographic data of patients were described using percentages, medians, and ranges. The SARS-CoV-2-IgG spike antibody levels were recorded as medians (ranges) and compared using the Mann–Whitney U test and Kruskal–Wallis H test. Pearson’s χ^2^ test and Fisher’s exact test were used to compare frequencies between groups. To assess the predictive factors associated with effective neutralization (SARS-CoV-2 IgG titers ≥ 4160 AU/mL), stepwise logistic regression multivariate analysis was used to analyze the co-variants with *p* < 0.1 in the univariate analysis. The odds ratios (ORs) of predictive factors are reported with 95% confidence intervals (CIs). Statistical analyses were performed using SPSS version 22 (IBM SPSS Statistics, IBM Corporation, Armonk, NY, USA). All statistical analyses were based on two-sided hypothesis tests, with statistical significance determined at *p* < 0.05.

## 3. Results

### 3.1. Patient Characteristics

From 1 January 2022 to 31 October 2022, a total of 68 patients with hematologic malignancies and 68 age- and gender-matched healthy control subjects were included in this study. Patient demographic data, disease characteristics, vaccination types, and baseline immunoglobulin levels are summarized in Table 1. The median age of the patients was 59 years (range, 26–78 years), and 32 (47.1%) were male. Thirty-six patients (52.9%) had a history of lymphoma, fifteen (22.1%) had chronic myeloid leukemia, nine (13.2%) had acute leukemia, and eight (11.8%) had multiple myeloma. While there were no statistically significant differences in Charlson comorbidity index values between the different cancer types, patients with lymphoma and multiple myeloma were reported to be older and to have more comorbidities compared to those with chronic myeloid leukemia and acute leukemia.

Forty-six patients (67.6%) had active cancer status, and forty patients (58.8%) had received active anticancer therapy within six months of vaccination. Among the actively treated patients, 14 (35.0%) had recent exposure to intravenous chemotherapy, 22 (55.0%) to corticosteroids, 8 (20.0%) to immunomodulatory drugs (IMiDs), 16 (40.0%) to BCR-ABL1 tyrosine kinase inhibitor (TKI), and 12 (30.0%) to B-cell-targeted agents, including anti-CD20 monoclonal antibodies, BTK inhibitors, and PI3K inhibitors. Seven (10.3%) patients underwent autologous or allogenic stem cell transplantation at least 12 months preceding vaccination. About two thirds (67.6%) of patients received the mRNA-based primary two-dose vaccination, which was followed by the AZ-based (25.0%), MVC-based (4.4%), and mixed vaccines (2.9%). The most used booster doses were mRNA vaccines, accounting for 86.8% of patients. There were no statistically significant differences in vaccine types between groups.

### 3.2. Serological Responses

**Figure 1:**
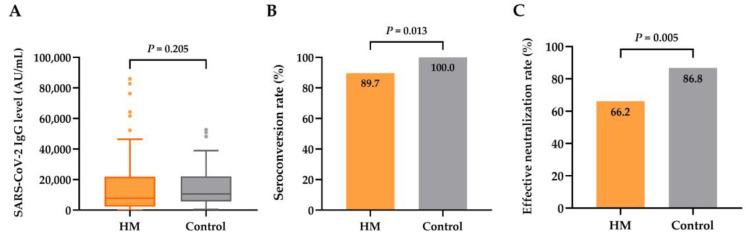
Serological responses to booster vaccines among patients with hematologic malignancies (HMs) (*n* = 68) and sex- and age-matched healthy control subjects (*n* = 68). (**A**) SARS-CoV-2-IgG spike antibody levels in patients and healthy controls. (**B**) Seroconversion rate in patients and healthy controls. (**C**) Rate of effective neutralization (SARS-CoV-2 IgG level ≥ 4160 AU/mL) in patients and healthy controls.

The median SARS-CoV-2-IgG spike antibody level was 7758.6 AU/mL (range, 0.0–85,943.0 AU/mL) in all patients, corresponding to 1101.8 BAU/mL (range, 0.0–12,203.9 BAU/mL). The majority of the patients (61 of 68, 89.7%) had positive antibody responses after booster vaccines. In contrast, seven patients (10.3%) with actively treated lymphoma failed to attain seroconversion despite the administration of booster doses. Forty-five patients (66.2%) achieved effective neutralizing immunity (anti-spike IgG level ≥ 4160 AU/mL). The median time from the booster dose to serology testing was 57 days. A sex- and age-matched analysis comparing the serological responses in 68 patients with hematologic malignancies (median age, 59 years; range, 26–78 years) and 68 age- and gender-matched healthy control subjects (median age, 59.5 years; range: 25–78 years) revealed comparable antibody titers in patients with hematologic malignancies (median of 7758.6 AU/mL (range, 0.0–85,943.0 AU/mL) vs. 10,647.0 AU/mL (range, 493.0–52,708.5 AU/mL), respectively; *p* = 0.205) but significantly reduced rates of seroconversion (89.7% vs. 100.0%, respectively; *p* = 0.013) and effective neutralization (66.2% vs. 86.8%, respectively; *p* = 0.005) (Figure 1A–C).

**Figure 2:**
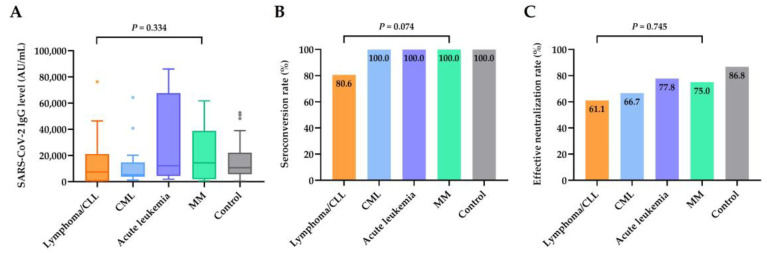
Serological responses of patients with different types of hematologic malignancies (*n* = 68). (**A**) SARS-CoV-2-IgG spike antibody levels in patients with lymphoma and chronic lymphocytic leukemia (CLL) (*n* = 36), chronic myeloid leukemia (CML) (*n* = 15), acute leukemia (*n* = 9), multiple myeloma (MM) (*n* = 8), and in healthy controls (*n* = 68). (**B**) Seroconversion rate in patients with different types of hematologic malignancies and healthy controls. (**C**) Rate of effective neutralization (SARS-CoV-2 IgG level ≥ 4160 AU/mL) in patients with different types of hematologic malignancies and healthy controls.

**Figure 3:**
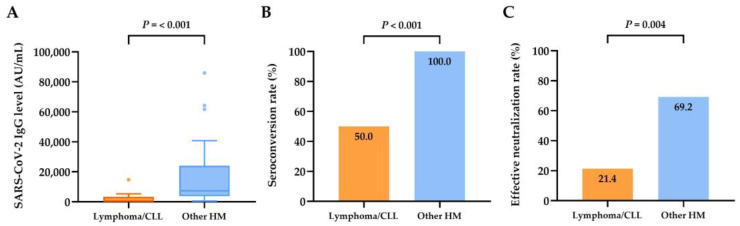
Serological responses of patients with actively treated hematologic malignancies (*n* = 40). (**A**) SARS-CoV-2-IgG spike antibody levels in patients with actively treated lymphoma/chronic lymphocytic leukemia (CLL) (*n* = 14) and other hematologic malignancies (HMs) (*n* = 26). (**B**) Seroconversion rate in patients with actively treated lymphoma/CLL (*n* = 14) and other HMs (*n* = 26). (**C**) Rate of effective neutralization (SARS-CoV-2 IgG level ≥ 4160 AU/mL) in patients with actively treated lymphoma/CLL (*n* = 14) and other HMs (*n* = 26).

Among the different types of hematologic malignancies, there were no statistical differences in antibody levels, rates of seroconversion, or effective neutralization (Figure 2A–C). Focusing on the actively treated patients, patients with lymphoma had a significantly lower median antibody level (median, 172.6 AU/mL; range, 0.0–14,765.7 AU/mL) and a markedly reduced seroconversion rate (7 of 14 (50.0%)) and rate of effective neutralization (3 of 14 (21.4%)) (Figure 3A–C) compared with patients with other hematologic malignancies.

**Figure 4:**
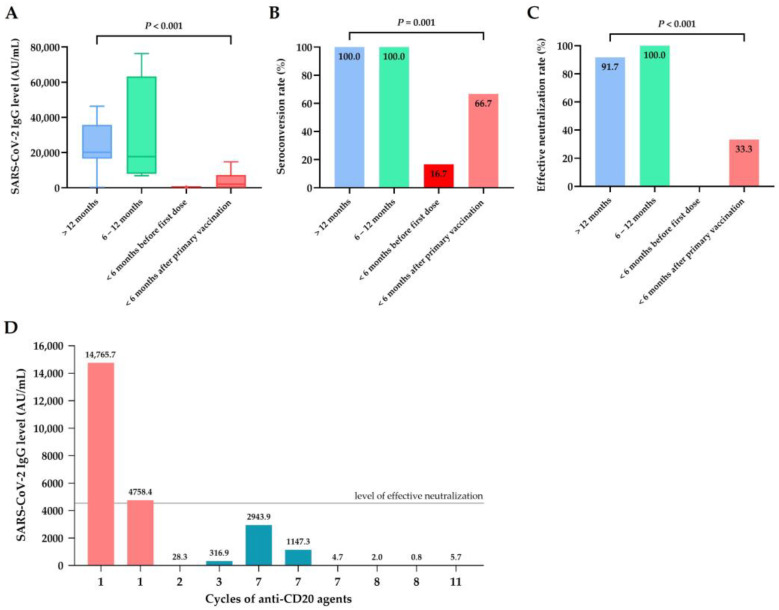
Serological responses of lymphoma patients receiving B-cell-targeted agents at different time points (*n* = 28). (**A**) SARS-CoV-2-IgG spike antibody levels in patients receiving B-cell-targeted agents at different time points: more than 12 months before the first vaccination (*n* = 12), 6–12 months before the first vaccination (*n* = 4), less than 6 months before the first vaccination (*n* = 6), and less than 6 months after primary vaccination. (**B**) Seroconversion rate at different time points. (**C**) Rate of effective neutralization (SARS-CoV-2 IgG level ≥ 4160 AU/mL) at different time points. (**D**) SARS-CoV-2-IgG spike antibody levels in patients receiving different cycles of anti-CD20 monoclonal antibodies within six months of vaccination (each bar indicates one lymphoma patient, *n* = 10).

Among the 28 lymphoma patients with exposure to B-cell-targeted agents, the rates of seroconversion (5 of 12 (41.7%)) and effective antibody neutralization (2 of 12 (16.7%)) were low in actively treated patients, whereas substantially better responses were observed in patients treated with B-cell-targeted agents at least six months from vaccination. Furthermore, patients receiving active treatment prior to the first vaccination exhibited an inferior response when compared to those who underwent therapy after completing primary vaccination (Figure 4A–C). None of the patients exposed to more than one cycle of anti-CD20 monoclonal antibodies during vaccination had effective neutralizing immunity (Figure 4D). One patient treated with ibrutinib and one with copanlisib at the time of vaccination failed to attain seroconversion despite completion of the booster dose.

### 3.3. Predictive Factors Associated with Effective Antibody Neutralization

In a univariate analysis (Table 2), the variables found to be significantly associated with effective antibody neutralization included lack of active B-cell-targeted agents, lack of active intravenous chemotherapy at the time of vaccination, and higher serum immunoglobulin levels fully meeting the cutoffs of IgG ≥ 550 mg/dL, IgA ≥ 80 mg/dL, and IgM ≥ 40 mg/dL. Age, gender, comorbidities, vaccine type(s), cancer status, TKI, and IMiDs had no statistically significant correlation with serological responses to vaccination.

In a stepwise multivariate analysis (Table 2), the independent predictors of antibody responses were confined to a lack of active B-cell-targeted agents with an OR of 15.177 (95% CI, 2.737–84.168; *p* = 0.002) and higher immunoglobulin levels (IgG ≥ 550 mg/dL, IgA ≥ 80 mg/dL, and IgM ≥ 40 mg/dL) with an OR of 4.375 (95% CI, 1.299–14.731; *p* = 0.017). Most patients (34 of 43 (79.1%)) with serum immunoglobulin levels fully meeting the cutoffs of IgG ≥ 550 mg/dL, IgA ≥ 80 mg/dL, and IgM ≥ 40 mg/dL achieved effective neutralizing immunity.

## 4. Discussion

This prospective study evaluated serological responses and potential predictive markers for effective neutralization of booster COVID-19 vaccination in Taiwanese patients with hematologic malignancies. Regardless of the type(s) of vaccines, nearly 90% of the patients in our cohort achieved seroconversion after booster doses, surpassing the seroconversion rate of 67.7% following primary two-dose vaccination documented in the previous meta-analysis [9]. This finding demonstrates the benefit of booster vaccination for patients with hematologic malignancies in enhancing immunogenicity against SARS-CoV-2. Though the serological response improved after the booster dose, patients with hematologic malignancies still had notably lower percentages of seroconversion and effective neutralization in comparison with healthy individuals. Despite the limited number of participants, postbooster seroconversion rates in our study were quite similar to outcomes reported in a larger Japanese cohort [23]. In line with prior studies [19,23,24], patients with actively treated lymphoid malignancies in our cohort had the lowest booster-induced antibody titers compared to patients with myeloid malignancies and plasma cell dyscrasias.

In our study, patients exposed to active B-cell-targeted agents, including anti-CD20 monoclonal antibodies, BTK inhibitors, and PI3K inhibitors, attained suboptimal antibody responses even after receiving booster vaccines. Our data suggest that a minimum time interval of six months is required between the administration of B-cell-depleting agents and vaccination. This timing is consistent with the recommended vaccine schedule outlined in the guidelines from the 2017 European Conference on Infections in Leukaemia (ECIL 7) for inactivated influenza, pneumococcal, and other inactivated vaccines [25], whereas other authors have reported a suggested time interval of 12 months [26,27,28]. Given that rituximab has been reported to have a more pronounced impact on the response to primary than to recall antigens [29], the time sequence of COVID-19 vaccination and B-cell-targeted agents may influence serological responses. Our study revealed that patients who had completed primary vaccination preceding the treatment with B-cell-targeted agents achieved a higher rate of seroconversion than those who were vaccinated after the therapy. This result is aligned with a recent study conducted by Ikeda et al. [23], in which patients receiving B-cell-depletion therapy after the second vaccine all experienced seroconversion and sustained the response.

Other than active therapy resulting in ongoing B-cell depletion, we identified hypogammaglobulinemia (IgG < 550 mg/dL, IgA < 80 mg/dL, or IgM < 40 mg/dL) as an independent predictive marker associated with a lower percentage of effective neutralization. Since measurement of the SARS-CoV-2-IgG spike antibody level is not widely available in real-world settings, immunoglobulin levels could be interpreted as a surrogate for recovery of humoral immunity, which is particularly useful in patients with hematologic malignancies to determine the optimal timing for additional vaccination. Previous studies [27,30,31] have also found a strong correlation between B-cell counts and anti-SARS-CoV-2 antibody titers. Other potential predictive factors that have been reported in patients with hematologic malignancies include age, use of BCL2 inhibitors, use of JAK2 inhibitors, absolute lymphocyte count, and circulating CD4+, CD8+, and natural killer cell counts [7,8,9,23,32].

A longitudinal prospective study conducted by Levin et al. demonstrated a time-dependent decay of the humoral response following primary vaccination, particularly among patients with immunosuppression [33], which once again highlighted the necessity for booster vaccines. Distinct from waning humoral immunity, cellular immunity might play an important role in long-term protection against SARS-CoV-2. Several studies have reported that the acquisition of SARS-CoV-2-specific T cells was associated with lower disease severity and accelerated viral clearance of COVID-19 [34,35,36,37]. In terms of vaccine-induced cellular immunity, Jimenez et al. [38] found that SARS-CoV-2-specific T-cell responses after two-dose mRNA vaccines were preserved in 60% of seronegative lymphoma patients treated with anti-CD20 therapy. Additionally, previous studies showed that the booster dose tended to generate an emerging cellular response in patients with hematologic malignancies, whereas the humoral response remained impaired [39,40]. The correlation between vaccine-induced cellular response and COVID-19-related outcomes in cancer patients warrants further clarification.

Considering the immune dysregulation related to diseases and treatment modalities, patients with hematologic malignancies experience a more severe clinical course of COVID-19, accounting for approximately 40% of severe to critical illnesses and 9% of mortality even in the postvaccine era [6]. As noted in our study, patients treated with ongoing B-cell-directed therapy were less likely to mount a sufficient vaccine response. Further protective strategies, including a second booster vaccination and passive immunization using monoclonal antibodies, should be provided for those high-risk populations. A retrospective study conducted by Ollila et al. revealed that administration of tixagevimab/cilgavimab effectively lowered the risk of COVID-19 deaths in vaccine nonresponders [18]. It is important to note that prompt management with antivirals in combination with monoclonal antibodies was crucial in patients with hematologic malignancies to improve COVID-19-related outcomes [6,41]. Nevertheless, given the constant evolution of SARS-CoV-2, previously authorized therapies might not maintain efficacy against current circulating Omicron subvariants [42,43,44]. Consequently, identifying optimal prophylactic and therapeutic approaches for COVID-19 in patients with hematologic malignancies remains challenging.

Certain limitations of our study must be acknowledged. First, a relatively small number of patients were enrolled and no baseline screening of SARS-CoV-2 nucleocapsid antibody was performed to ensure prior infection. Second, we did not evaluate serological responses following the second vaccination to ascertain booster-induced seroconversion. Third, we only assessed the humoral response, but the cellular response is reported to play an important role in mitigating COVID-19. Finally, the COVID-19-related outcomes of patients in relation to vaccine responses were not reported in our study. Future prospective studies with a larger sample size are warranted to evaluate the postbooster cellular immunity in patients with hematologic malignancies.

## 5. Conclusions

Nearly 90% of patients with hematologic malignancies achieved seroconversion after booster vaccination, while patients with actively treated lymphoma had the lowest serological responses. Recent exposure to B-cell-targeted agents within six months of vaccination and hypogammaglobinemia were identified as negative predictive markers for effective neutralization.

## Figures and Tables

**Table 1:** Patient characteristics stratified by types of cancer (*n* = 68).

	Lymphoma and CLL (*n* = 36)	Chronic Myeloid Leukemia (*n* = 15)	Acute Leukemia (*n* = 9)	Multiple Myeloma (*n* = 8)	*p*-Value
Age, median (range), years	62 (26–78)	56 (27–71)	56 (27–71)	73.5 (52–75)	0.012 *
Male gender, *n* (%)	18 (50.0%)	5 (33.3%)	4 (44.4%)	5 (62.5%)	0.562
BMI, median (range), kg/m^2^	24.2 (18.1–35.0)	23.7 (19.3–33.2)	24.7 (20.5–30.9)	23.6 (16.5–30.0)	0.667
Comorbidities, *n* (%)	27 (75.0%)	5 (33.3%)	5 (55.6%)	5 (62.5%)	0.047 *
Hypertension	14 (38.9%)	2 (13.3%)	0 (0.0%)	3 (37.5%)	
Diabetes	7 (19.4%)	1 (6.7%)	3 (33.3%)	2 (25.0%)	
Chronic kidney disease	4 (11.1%)	2 (13.3%)	0 (0.0%)	4 (50.0%)	
Chronic liver disease ^1^	11 (30.6%)	1 (6.7%)	2 (22.2%)	0 (0.0%)	
Cardiovascular disease ^2^	0 (0.0%)	0 (0.0%)	0 (0.0%)	1 (12.5%)	
Cerebrovascular disease	1 (2.8%)	0 (0.0%)	0 (0.0%)	0 (0.0%)	
Peptic ulcer disease	5 (13.9%)	1 (6.7%)	0 (0.0%)	1 (12.5%)	
Charlson comorbidity index, median (range)	4 (2–10)	3 (2–5)	3 (2–5)	3 (1–7)	0.206
Active cancer, *n* (%)	21 (58.3%)	15 (100.0%)	2 (22.2%)	8 (100.0%)	<0.001 ***
Active treatment within 6 months, *n* (%)	14 (38.9%)	15 (100.0%)	3 (33.3%)	8 (100.0%)	<0.001 ***
IV chemotherapy	12 (33.3%)	0 (0.0%)	2 (22.2%)	0 (0.0%)	
B-cell-targeted agent ^3^	12 (33.3%)	0 (0.0%)	0 (0.0%)	0 (0.0%)	
Immunomodulatory drug	0 (0.0%)	0 (0.0%)	0 (0.0%)	8 (100.0%)	
BCR-ABL TKI	0 (0.0%)	15 (100.0%)	1 (11.1%)	0 (0.0%)	
Anti-CD38 monoclonal antibodies	0 (0.0%)	0 (0.0%)	0 (0.0%)	2 (25.0%)	
Glucocorticoids	12 (33.3%)	1 (6.7%)	1 (11.1%)	8 (100.0%)	
HSCT, *n* (%)	2 (5.6%)	0 (0.0%)	2 (22.2%)	3 (37.5%)	0.015 *
First and Second vaccinations, *n* (%)					0.913
AZ-based	9 (25.0%)	4 (26.7%)	2 (22.2%)	2 (25.0%)	
mRNA-based	24 (66.7%)	10 (66.7%)	7 (77.8%)	5 (62.5%)	
MVC-based	1 (2.8%)	1 (6.7%)	0 (0.0%)	1 (12.5%)	
Mixed ^4^	2 (5.6%)	0 (0.0%)	0 (0.0%)	0 (0.0%)	
Booster vaccination, *n* (%)					0.503
mRNA	33 (91.7%)	12 (80.0%)	8 (88.9%)	6 (75.0%)	
Medigen	3 (5.6%)	3 (20.0%)	1 (11.1%)	2 (25.0%)	

CLL, chronic lymphocytic leukemia; BMI, body mass index; IV, intravenous; TKI, tyrosine kinase inhibitor; HSCT, hematopoietic stem cell transplantation; AZ, AstraZeneca; mRNA, messenger RNA; MVC, Medigen COVID-19 vaccine. * and ***  =  *p*-value  <  0.05 and 0.001, respectively. ^1^ Chronic liver disease: chronic hepatitis C, chronic hepatitis B, and cirrhosis; ^2^ cardiovascular disease: myocardial infarction, heart failure, and peripheral vascular disease; ^3^ B-cell-targeted agent: anti-CD20 monoclonal antibody, Bruton tyrosine kinase (BTK) inhibitor, and phosphatidylinositol-3 kinase (PI3K) inhibitor; ^4^ mixed vaccination: AZ–Moderna (*n* = 1), AZ–BioNTech (*n* = 1).

**Table 2:** Factors associated with SARS-CoV2-IgG spike antibody level with effective neutralization (≥ 4160 AU/mL).

Variable	Univariate OR (95% CI)	*p*-Value	Multivariate OR (95% CI)	*p*-Value
Age < 65 years	2.030 (0.722–5.704)	0.179		
Female gender	1.779 (0.645–4.907)	0.266		
Without hypertension	1.648 (0.552–4.926)	0.371		
Without diabetes	0.842 (0.229–3.097)	0.796		
Without chronic kidney disease	3.618 (0.905–14.463)	0.069	1.939 (0.353–10.645)	0.446
Without chronic liver disease	0.737 (0.203–2.669)	0.642		
Charlson comorbidity index < 4	1.488 (0.536–4.131)	0.446		
mRNA-based first and second vaccines	1.582 (0.550–4.553)	0.395		
Inactive cancer	3.167 (0.924–10.857)	0.067	0.985 (0.229–4.235)	0.984
Lack of active B-cell-targeted agent	16.538 (3.208–85.261)	0.001 **	15.177 (2.737–84.168)	0.002 **
Lack of active IV chemotherapy	7.885 (2.113–29.418)	0.002 **	2.053 (0.327–12.876)	0.443
Lack of active glucocorticoids	2.833 (0.978–8.209)	0.055	0.502 (0.099–2.550)	0.406
BCR-ABL TKI	0.917 (0.290–2.902)	0.882		
Immunomodulatory drug	1.615 (0.299–8.719)	0.577		
IgG ≥550, IgA ≥80, IgM ≥40 mg/dL	4.808 (1.635–14.139)	0.004 **	4.375 (1.299–14.731)	0.017 *

mRNA, messenger RNA; IV, intravenous; TKI, tyrosine kinase inhibitor. * and **  =  *p*-value  <  0.05 and 0.01, respectively.

## Data Availability

Inquiries about data availability should be directed to the corresponding author due to the privacy of patients’ results.
